# The Role of Angiotensin Antagonism in Coronary Plaque Regression: Insights from the Glagovian Model

**DOI:** 10.1155/2021/8887248

**Published:** 2021-04-07

**Authors:** Abdul H. Alkatiri, Doni Firman, Amir A. Alkatiri, Paskalis I. Suryajaya, Albert Sudharsono

**Affiliations:** ^1^Department of Cardiology and Vascular Medicine, Hasanuddin University, 90245, Indonesia; ^2^Department of Cardiology and Vascular Medicine, Faculty of Medicine, Universitas Indonesia, National Cardiovascular Center Harapan Kita, Jakarta 11420, Indonesia

## Abstract

The benefit of antagonizing the effect of the renin angiotensin aldosterone system (RAAS), notably by the use of angiotensin-converting enzyme inhibitor (ACEi) and angiotensin II type 1 receptor blocker (ARB) for coronary artery disease (CAD), has been demonstrated in multiple studies, which may be attributed to their ability to inhibit the deleterious effect of RAAS to the cardiovascular system. It is well known that angiotensin II (Ang II) plays a vital role in atheromatous plaque formation and progression through multiple pathways, including inflammatory and arterial remodeling aspects. Significant coronary atheromatous plaque regression has been previously demonstrated in various studies using statin agents. Similar results have been reported in different studies using angiotensin inhibitor agents, notably ARB agents. Analysis from various trials utilizing ARB showed a significant plaque regression using olmesartan and telmisartan as evaluated by IVUS studies. In contrary, the use of ACEi did not demonstrated significant plaque regression, which may be attributed to the heavy plaque calcification in respective studies. On this review, we aim to present the basic mechanism on the role of RAAS in plaque modulation and its arterial remodeling aspect, which is then integrated with the clinical evidence based on the available intravascular ultrasonography (IVUS) studies on coronary arteries.

## 1. Introduction

Atheromatous plaque has been traditionally viewed as a unidirectional protrusion of the atheroma into the lumen, resulting in a lumen stenosis. It was not until 1987 when Glagov et al. demonstrated that rather than an initial phase of the atherosclerotic disease, this protrusion is a final result of a long-term compromised compensation process of the vascular structure on maintaining the lumen area patency in the presence of growing atheroma within the vascular wall structure [1]. They demonstrated that the vascular luminal area was in fact unaffected by the atheromatous plaque growth until the lesion exceeded 40% area stenosis, a phenomenon that was closely related to the vascular wall remodeling. Since then, the glagovian model has been widely used to study the effect of various pharmacological agents on the atheromatous plaque progression and the remodeling process of the surrounding vascular wall layers, namely, with the utilization of intravascular ultrasound (IVUS). These visionary studies facilitate the emerging concept of the dynamic bidirectional progression of atheromatous plaque, namely, the introduction of plaque regression concept.

Angiotensin inhibitors such as ACE inhibitor (ACEi) and angiotensin II type 1 receptor blocker (ARB) have been proven beneficial for subjects with coronary artery disease (CAD), which may be attributed to their ability to inhibit the deleterious effect of RAAS [2]. The beneficial effect of angiotensin inhibitors, notably ARB, on cardiovascular outcome may also be attributed to its pleiotropic effect on the atheromatous plaque progression and its anti-inflammatory effect [3–5]. Moreover, long-term RAAS activity has been related with the atheromatous plaque formation and progression [6]. Various trials have been conducted to demonstrate the effect of ACEi and ARB on coronary atherosclerotic plaque modulation by IVUS study. The purpose of this review is to present the proposed mechanism and the current evidence regarding the effect of angiotensin inhibitor agents on the coronary atheromatous plaque.

## 2. The Concept of Arterial Remodeling

By conducting a postmortem study on human coronary arteries, Glagov et al. demonstrate that a protrusion of plaque into the arterial lumen was in fact a product of a failing compensatory process by the arterial remodeling [1]. Thus, even an angiographically mild stenosis might be a result of a big atheromatous plaque embedded in the arterial wall that exceeded the threshold of arterial wall remodeling. Arterial remodeling itself is defined as alterations in the structure and function of the vascular wall. In the early process of atherosclerosis, an inflammation process took place in the intima as a result of the accumulation of lipids in the plaque. The progression of this plaque results in compensatory dilatation of the arterial wall. This outward direction of remodeling is defined as positive remodeling, a change of the arterial wall structure that was characterized by an expansion of external elastic membrane (EEM) to accommodate the atheromatous plaque progression in exchange of maintaining lumen patency. This process is majorly moderated by nitric oxide and matrix metalloproteinase (MMP) [7, 8]. The plaque area which resides in the EEM area (commonly termed as the plaque burden) will then start to result in lumen stenosis when the threshold of 40% is surpassed. Since the natural history of the plaque is to progress within the EEM area, positive remodeling is also commonly defined as an expansion of the EEM area. This period of plaque progression is commonly found in subjects with unstable angina, since the plaque build-up dominates over the fibrotic changes of the EEM, producing a vulnerable plaque, prone for rupture [9]. This is confirmed by an IVUS study by Schoenhagen et al., where they demonstrate that positive remodeling was found largely in unstable plaque compared to the stable plaque [10].

**Figure 1:**
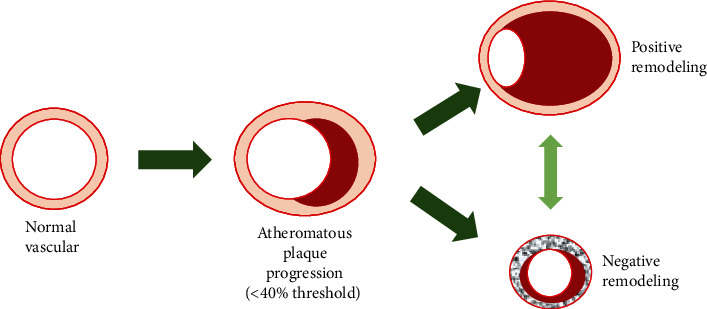
Stages of vascular remodeling. Atheromatous plaque progression will not affect the lumen area until the 40% threshold has been surpassed. The EEM may compensate the plaque build-up with outward remodeling (positive remodeling). Fibrotic changes of the EEM area may result in the shrinkage of the vessel area, leading to reduced vessel area (negative remodeling).

The arterial remodeling process may also progress in a different direction, notably toward the arterial lumen, which is known as negative remodeling. In contrast to its counterpart, negative remodeling is characterized by a shrinkage of the EEM area attributed to the increased fibrotic changes [11]. These changes are commonly thought as an advanced phase of atherosclerosis in which the long-term fibrotic change results in a shrinkage EEM morphology and luminal stenosis [12, 13]. This period is commonly related to stable angina, since the fibrotic changes result in a more stable plaque and fibrous cap, reducing the frailty of the plaque [14] Hence, the two types of arterial remodeling may be viewed continuum, with positive remodeling occurring in the initial stage of atherosclerosis and negative remodeling as the advanced firm stage of atherosclerosis (see Figure 1).

## 3. Basic Mechanism of Arterial Remodeling

Histologic studies of atherosclerotic disease artery provide a proof that the remodeling process is a product of pathological hemodynamic changes and inflammatory process leading into an inappropriate remodeling of the arterial wall structure [15]. Disruption of local physiological hemodynamic factors has been shown to be a key point that initiates the remodeling process [16] (i.e., vessel geometry, blood flow, shear stress, and wall tensile stress). There were three central process known as the basic of arterial remodeling; vascular smooth muscle cell (VSMC) proliferation and phenotype switching, elastin degradation, and vascular calcification [17].

### 3.1. VSMC Proliferation and Phenotype Switching

The arterial wall comprises of multiple radial layers consisting of internal membrane, basement membrane, internal elastic membrane, medial membrane, external elastic membrane, and adventitia. In atherosclerosis, extracellular matrix (ECM) structural change is the most notable process seen, mainly on the medial membrane and EEM. These two layers are normally consisting of elastic fibers, glycoproteins, integrins, and smooth muscle cell (SMC). Remodeling process results in a disruption of these structures, leading to fibrotic changes [16].

SMC mainly has a contractility phenotype, which is responsible for the modulation of vascular tone. But under some circumstances, SMC possesses the ability to switch to another phenotype, namely, the synthetic phenotype, which can be further categorized into three distinct features: migratory-proliferative phenotype, secretory phenotype, and osteogenic phenotype [18]. This phenotype-switching ability results in a gross structural change of the arterial wall, depending on the initiating insults [19]. Common insults comprise of systemic and/or local inflammatory process, oxidative injury, or local hemodynamic profile (i.e., wall stress) [15].

Migratory-proliferative phenotype is characterized with certain SMC cytoskeletal structural changes that enable it to migrate to the source of initiating stimuli. A lamellipodia can be seen projecting from the SMC structure and helps it move across the adjacent tissue [20]. In atherosclerosis, this phenotype is responsible for the migration of SMC to the internal membrane. To facilitate this migratory process, a detachment of SMC from its surrounding tissues is mandatory, which is mediated by MMP activity. The inhibition of MMP has been related to the inhibition of SMC migration, particularly after vascular injury [21]. Overall, the migratory and proliferative events seen in vascular remodeling re mediated by angiotensin II (Ang II) activity [22].

### 3.2. Elastin Degradation and Vascular Calcification

Elastin is a major component of the medial membrane. Unlike those seen in infants, elastin degradation in adults does not followed by its regeneration, but by a collagen production [23]. As a result, elastin degradation by MMP will lead to the increase of arterial wall stiffness, which is commonly seen after vascular injury. Ang II will accentuate the activity of MMP-2 and MMP-9 leading to the elastin degradation [24].

Vascular calcification is a net result of the imbalance between factors that induce and inhibit calcium deposition in the tissue. Physiologically, elastin has a calcium-binding capacity, supporting the calcification process. MMP–mediated elastin degradation will initiate the progression of vascular calcification. Although the exact mechanism remains unknown, it is postulated that elastin degradation will accentuate its calcium affinity [25]. Other mechanism for the increased vascular calcification includes the attenuation of matrix GLA protein (MGP) activity which normally will inhibit the bone morphogenetic protein-2 (BMP-2). This results in the increased osteogenic phenotype of SMC [26].

## 4. The Role of the Renin Angiotensin Aldosterone System in Atheromatous Plaque Progression

**Figure 2:**
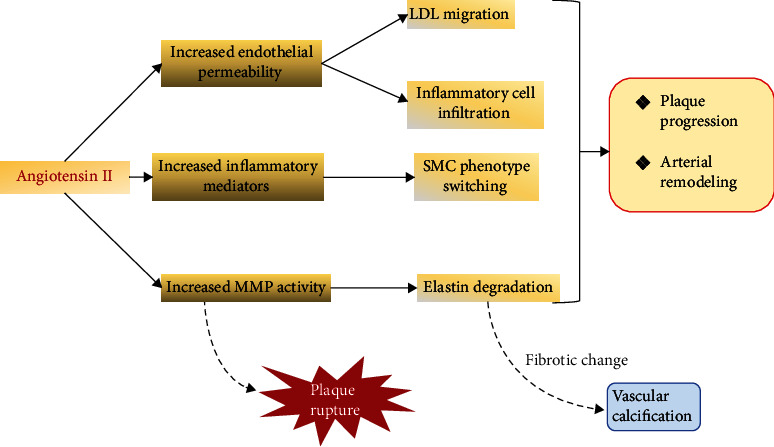
Angiotensin II role in atheromatous plaque modulation (LDL: low-density lipoprotein; MMP: matrix metalloproteinase; SMC: smooth muscle cell).

Endothelial cells injury has been known as the initial process leading to atheromatous plaque formation and progression. Various stimuli including hyperlipidemia, hypertension, hyperglycemia, free radicals, and shear stress have been shown to induce the injury process and lead to endothelial dysfunction [27, 28]. This will increase the endothelial permeability, permitting low-density lipoprotein and inflammatory cell accumulation within the medial membrane, and trigger the extensive inflammatory process within the arterial wall [29]. The inflammatory process triggers proinflammatory cytokine and certain growth factors that will promote the migratory-proliferative phenotype of SMC, resulting to its migration towards the internal membrane and the forming of fibrous cap [30, 31] (see Figure 2).

RAAS activity is associated with the increase of the vascular inflammatory response, which promotes the atheromatous plaque progression. Ang II may recruit inflammatory cells and initiate the triggering event in atheromatous plaque progression [32, 33]. These inflammatory cells may paradoxically produce Ang II that accentuate the inflammatory response, further extending the plaque progression by promoting migratory-proliferative activity of SMC and lipid core growth [33].

## 5. Atheromatous Plaque Regression

Atheromatous plaque was previously viewed as a one-way trip towards its progression, but further studies conclude that it is rather a dynamic process, leading to the new understanding on the possibility of plaque regression. The mechanism of plaque regression itself is not synonymous to the reversal of plaque progression and process, but rather a breakdown process consisting of the removal of lipid component, the removal of macrophage within plaque, and the reversal of the pathological phenotype switching [29]. Nevertheless, despite wide and vigorous studies on the plaque regression, animal studies shown that a successful total plaque regression has not been able to be achieved [34].

Removal of lipid component within atheromatous plaque has been known as reverse cholesterol transport (RCT), a complex lipid transport system with high-density lipoprotein (HDL) as its main element [35]. The main goal of RCT is the transport of lipid components from peripheral tissues to the liver. This is a very crucial step, since lipid is the core and main constituent of atheromatous plaque. A successful transport of cholesterol by the HDL to the liver will then followed by its removal through the hepatobiliary route [29].

Since macrophage plays an important role in the initial process of plaque formation, its removal within plaque is also mandatory for plaque regression. Animal studies show that macrophage removal is consistent with the plaque regression [34]. This is an active process, in which the macrophage may migrate from the plaque, leaving for regional lymphoid tissues [36]. The migration of macrophage will then mediate the reduction of the inflammatory process, within plaque, removing the deleterious effect of inflammation within the plaque and arterial wall layers. Together with the constant removal of the lipid component, this will promote the reversal of pathological SMC phenotype switching, leading to a better SMC affinity to HDL which will potentiate the lipid removal [34]. Statin therapy has been shown in numerous studies to effectively increase the serum HDL level, implying its role in plaque regression by supporting the RCT mechanism and also its pleiotropic anti-inflammatory effect. Since Ang II accentuates the inflammatory process and MMP-mediated arterial wall remodeling, the use of angiotensin inhibitors may benefit the process of plaque regression.

## 6. The Clinical Effect of Angiotensin Inhibitors on Atheromatous Plaque Regression

Intravascular ultrasound (IVUS) provide an effective measure to evaluate the coronary plaque and its surrounding. Various studies on the effect of pharmacological agent on plaque progression/regression utilize IVUS. Majority of the published studies on plaque regression comprise of statin therapy, while there are only a few of those studying angiotensin inhibitor. These studies are summarized in Table 1.

### 6.1. Angiotensin II Type 1 Receptor Blocker

**Figure 3:**
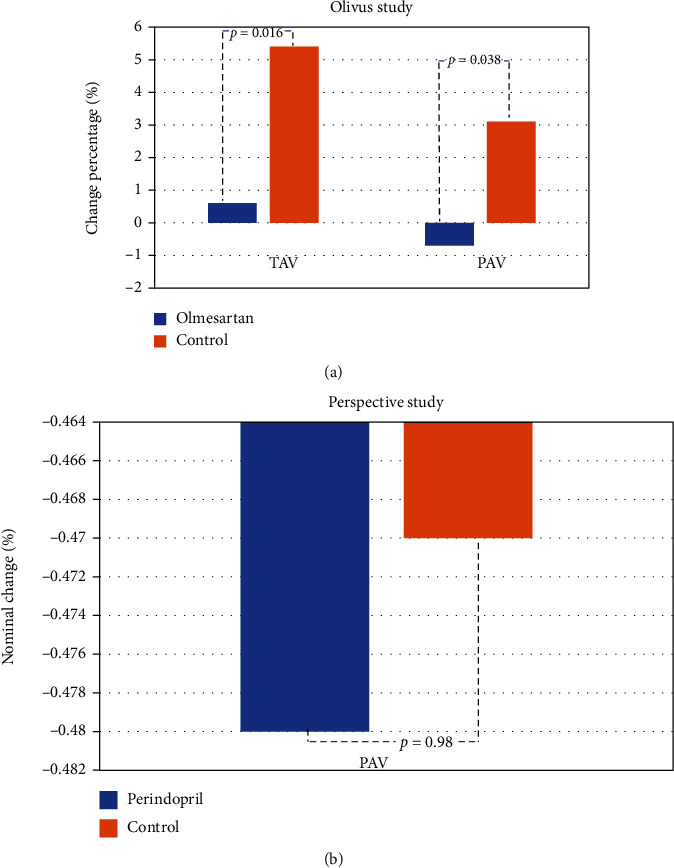
(a) Change percentage of total atheroma volume (TAV) and percentage atheroma volume (PAV) in OLIVUS study in 14-months follow-up. Olmesartan significantly demonstrates a greater change in both parameters. (b) PERSPECTIVE study demonstrated no significant difference in PAV change between the perindopril and control group.

The Impact of Olmesartan on Progression of Coronary Atherosclerosis: Evaluation by Intravascular Ultrasound (OLIVUS) study was of the biggest trial in the field of evaluating the effect of angiotensin inhibitor on the coronary plaque composition, consisting of 247 stable angina subjects randomized for olmesartan (10, 20, or 40 mg) vs. placebo in addition to conventional therapy; i.e., beta blockers (BB), calcium channel blockers (CCB), statins, nitrate, and antiglycemic [37]. The endpoints were total atheroma volume (TAV), percent atheroma volume (PAV), and vessel volume, which was obtained by serial IVUS study on the coronary artery. In the 14 months follow-up, the olmesartan group showed a better outcome in terms of greater nominal change in TAV compared to placebo (-2.6 vs. 7.1, *p* = 0.011). Similar result was obtained for the change percentage of TAV (0.6 vs. 5.4, *p* = 0.016) and PAV (-0.7 vs. 3.1, *p* = 0.038) (Figure 3). This study demonstrates the promising utility of olmesartan in reducing the plaque progression on clinically stable CAD.

Another ARB agent that was studied on the plaque regression was valsartan and telmisartan. A prospective, randomized, multicenter study by Ishii et al. aims to compare the effect of olmesartan (20 mg) vs. valsartan (80 mg) in addition to other conventional therapy in 94 stable angina subjects undergoing PCI [38]. The endpoints were TAV, PAV, vessel diameter, and lumen diameter on serial IVUS study in 6 months follow-up. Both groups demonstrated a significant reduction of plaque volume compared to baseline, but there was no significant difference in the change of TAV (-4.7 vs. -4.8, *p* = 0.96) and PAV (-2.5 vs. -2.8, *p* = 0.84), implying that both ARB agents share the same efficacy on plaque regression.

Yamaguchi et al. perform a prospective randomized study on 50 stable angina subjects with hypertension to compare telmisartan (80 mg) vs. other antihypertensive agents comprising of BB and CCB [41]. They conduct serial IVUS study within 6 months follow-up period and compared the TAV, PAV, and other vascular wall indices (i.e., vessel area, lumen area, fibrous volume, and calcified volume). The addition of telmisartan to the conventional statin therapy resulted in a greater PAV change compared to other antihypertensive agents (-6.5 vs. -1.1, *p* = 0.01). Interestingly, this study also evaluates the inflammatory parameters, namely, MMP, tumor necrosis factor alpha, and high sensitivity C-reactive protein between the two groups. The significant reduction of these inflammatory parameters compared to baseline levels was only demonstrated in the telmisartan group, implying pleiotropic anti-inflammatory effect as the potential mechanism of the PAV change.

### 6.2. Angiotensin-Converting Enzyme Inhibitor

The study on the effect of ACEi on the atheromatous plaque dates back before the OLIVUS study. The Perindopril's Prospective Effect on Coronary Atherosclerosis by Intravascular Ultrasound Evaluation (PERSPECTIVE) study was conducted in 2007 to evaluate the long-term effect of perindopril on the coronary atheromatous plaque progression by IVUS study [39]. In this prospective randomized study, the investigators aim to compare the effect of perindopril (8 mg) vs. placebo in addition to conventional therapy on 118 stable CAD subjects. The endpoints include PAV as well as lumen, vessel, and plaque cross-sectional area by serial IVUS study. After 3 years follow-up, they found no significant difference of PAV change between groups (-0.48 vs. -0.47, *p* = 0.98) (see Figure 3). Nevertheless, a posthoc analysis demonstrated that perindopril was associated with a negative remodeling pattern of the arterial wall without significant reduction of the lumen area, which implies a more stable plaque characteristic [42].

Another study on the use of ACEi was the one conducted by Han et al., where they aim to compare the effect of rosuvastatin (20 mg) alone vs. rosuvastatin (20 mg) plus ramipril (10 mg) on 40 stable CAD subjects. A 9–12 months follow-up period was done with a serial IVUS study [40]. Similar to the PERSPECTIVE study result, they found that the ACE inhibitor failed to show a significant difference on TAV and PAV change percentage compared to the control group (TAV *p* = 0.37, PAV *p* = 0.74).

## 7. Possible Mechanism on the Different Results between ACEi and ARB Studies

Based on the clinical data presented in this review, ARB usage was significantly related to the modulation of atheromatous plaque progression and regression. Olmesartan, valsartan, and telmisartan use all result in a significant plaque composition modulation, notably a reduction in progression, and even a regression. These results are consistent with the key role of Ang II on the basic pathophysiology of atheromatous plaque formation and progression, notably on the maintenance of inflammatory loop and arterial wall remodeling.

The different results demonstrated in studies consisted of ACEi may be hypothetically related to a class effect between ACEi. Perindopril demonstrated a greater eNOS expression compared to trandolapril, ramipril, and enalapril [43]. Moreover, in vitro study showed a difference of tissue ACE binding ability between several ACEi agents [44]. Other studies attribute the variable vascular effects between ACEi agents to the difference of tissue ACE potency [45].

Other potential reason for the lack of effect of ACEi on plaque regression may be attributed by the heterogenous plaque characteristic of the study, notably the plaque calcification status. On a subanalysis of PERSPECTIVE study, plaque regression with perindopril was demonstrated only on noncalcified plaque and represents a potential benefit on early stages of atherosclerotic disease [46]. In contrary, no significant plaque modulation activity was observed on moderately calcified plaque, which may imply that the use of ACEi may be limited to certain plaque on early stages without marked calcification [47].

## 8. Conclusions

Pharmacological agents that inhibit the angiotensin activity may modulate the plaque formation, progression, and even regression through a complex mechanism consisting of inflammation and arterial wall remodeling. RAAS, notably via Ang II activity, holds the key to the atheromatous plaque composition via inflammatory modulation, inducing arterial remodeling by MMP activity and accentuates the phenotype switching of SMC.

ARB usage demonstrated a significant modulation on plaque progression and regression as shown by multiple IVUS studies. Underlying mechanism for this beneficial effect seems to be closely related to the anti-inflammatory effect of ARB by blocking the Ang II activity.

## Figures and Tables

**Table 1:** Coronary plaque modulation in the trials with angiotensin inhibitors.

Clinical trial, year	Design (follow-up length)	Intervention	Subjects (no. of subjects)	Endpoints	Baseline		Follow-up		Nominal change	Change percentage of TAV and PAV (%)
					Total atheroma volume (mm^3^)	Percent atheroma volume (%)	Total atheroma volume (mm^3^)	Percent atheroma volume (%)		
OLIVUS [37] (2010)	Prospective randomized (14 months)	Olmesartan 10,20,or 40 mg/d vs. placebo (±BB, CCB, diuretic, nitrate, statin, antiglycemic)	Stable angina with native CAD (247 subjects)	TAV, PAV, vessel, volume, BP, MACE	230.2 ± 151.7 (OL) 208.8 ± 151.5 (placebo)	43.8 ± 10.2 (OL) 40.6 ± 10.8 (placebo)	227.6 ± 145.8 (OL) 215.9 ± 156.8 (placebo)	43.7 ± 10.4 (OL) 41.7 ± 11.5 (placebo)	TAV (mm^3^) -2.6 (OL) vs.7.1 (placebo) (*p* = 0.011) PAV (%) -0.1 (OM) vs. 1.1 (placebo) (*p* = 0.085)	TAV 0.6 (OL) vs.5.4 (placebo) (*p* = 0.016) PAV -0.7 (OM) vs. 3.1 (placebo) (*p* = 0.038)
PERSPECTIVE [39] (2007)	Prospective, double blind, randomized, multicentre (3 years)	Perindopril 8 mg/d vs. placebo (±BB, CCB, nitrate, lipid lowering agents, antiplatelet)	Stable CAD (history of MI, revascularization, angiographically significant coronary stenosis) without clinical evidence of HF (118 subjects)	CSA of vessel, lumen, and plaque; PAV	NA	41.29 ± 13.1 (PE) 39.01 ± 12.8 (placebo)	NA	40.81 ± 13.4 (PE) 38.55 ± 14.3 (placebo)	PAV (%) -0.48 (PE) vs. -0.47 (placebo) (*p* = 0.98)	NA
Ishii et al. [38] (2013)	Prospective, randomized, multicentre (6 months)	Olmesartan 20 mg/d vs. valsartan 80 mg/d (±BB, CCB, statins, antiglycemic)	Stable angina pectoris, already on statin for >8weeks before PCI (94 subjects)	TAV, PAV, vessel, and lumen volume	46.2 ± 24.1 (OL) 47.2 ± 32.7 (VAL)	43.4 ± 12.5 (OL) 40.6 ± 12.2 (VAL)	41.6 ± 21.1 (OL) 42.5 ± 30.2 (VAL)	38.2 ± 11.5 (OL) 40.6 ± 12.0 (VAL)	TAV -4.7 (OL) vs. -4.8 (VAL) (*p* = 0.96) PAV -2.5 (OL) vs. -2.8 (VAL) (*p* = 0.84)	NA
Han et al. [40] (2012)	Prospective, randomized (9-12 months)	Rosuvastatin 20 mg/d vs. rosuvastatin 20 mg/d + ramipril 10 mg/d (±BB, CCB, nitrate, antiplatelet, antiglycemic)	Stable intermediate de novo CAD without statin therapy within 2 months (40 subjects)	TAV, PAV, EEM volume, lipid parameter, biomarkers	68.8 ± 21.8 (ROS) 85.0 ± 22.5 (ROS + RAM)	46.2 ± 11.2 (ROS) 51.8 ± 10.5 (ROS + RAM)	63.4 ± 25.2 (ROS) 72.8 ± 20.3 (ROS + RAM)	44.3 ± 11.6 (ROS) 49.1 ± 10.1 (ROS + RAM)	NA	No significant difference of TAV and PAV change percentage between the two groups (*p* = 0.37, *p* = 0.74*p*, respectively)
Yamaguchi et al. [41] (2014)	Prospective, randomized (6 months)	Telmisartan 80 mg/d vs. other antihypertensive agents (CCB or BB)	Stable angina with HT, already on statin for >6 months (50 subjects)	TAV, PAV, vessel and lumen area, fibrous volume, calcified volume	21.0 ± 6.0 (TEL) 21.7 ± 11.4 (control)	38.4 ± 12.4 (TEL) 38.0 ± 11.0 (control)	20.8 ± 6.2 (TEL) 21.8 ± 10.5 (control)	32.8 ± 9.7 (TEL) 36.8 ± 12.1 (control)	NA	PAV -6.5 (TEL) vs. -1.1 (control) (*p* = 0.01)

## Data Availability

The data can be accessed in https://www.onlinejacc.org/content/55/10/976.abstracthttps://www.sciencedirect.com/science/article/abs/pii/S0002914913008990https://www.jstage.jst.go.jp/article/circj/advpub/0/advpub_CJ-13-0741/_article/-char/ja/https://www.sciencedirect.com/science/article/abs/pii/S0002914907006984https://www.sciencedirect.com/science/article/abs/pii/S016752731100060X.
